# Modern classification of neoplasms: reconciling differences between morphologic and molecular approaches

**DOI:** 10.1186/1471-2407-5-100

**Published:** 2005-08-10

**Authors:** Jules Berman

**Affiliations:** 1 U.S. National Cancer Institute, Cancer Diagnosis Program, Bethesda, USA

## Abstract

**Background:**

For over 150 years, pathologists have relied on histomorphology to classify and diagnose neoplasms. Their success has been stunning, permitting the accurate diagnosis of thousands of different types of neoplasms using only a microscope and a trained eye. In the past two decades, cancer genomics has challenged the supremacy of histomorphology by identifying genetic alterations shared by morphologically diverse tumors and by finding genetic features that distinguish subgroups of morphologically homogeneous tumors.

**Discussion:**

The Developmental Lineage Classification and Taxonomy of Neoplasms groups neoplasms by their embryologic origin. The putative value of this classification is based on the expectation that tumors of a common developmental lineage will share common metabolic pathways and common responses to drugs that target these pathways. The purpose of this manuscript is to show that grouping tumors according to their developmental lineage can reconcile certain fundamental discrepancies resulting from morphologic and molecular approaches to neoplasm classification.

In this study, six issues in tumor classification are described that exemplify the growing rift between morphologic and molecular approaches to tumor classification: 1) the morphologic separation between epithelial and non-epithelial tumors; 2) the grouping of tumors based on shared cellular functions; 3) the distinction between germ cell tumors and pluripotent tumors of non-germ cell origin; 4) the distinction between tumors that have lost their differentiation and tumors that arise from uncommitted stem cells; 5) the molecular properties shared by morphologically disparate tumors that have a common developmental lineage, and 6) the problem of re-classifying morphologically identical but clinically distinct subsets of tumors. The discussion of these issues in the context of describing different methods of tumor classification is intended to underscore the clinical value of a robust tumor classification.

**Summary:**

A classification of neoplasms should guide the rational design and selection of a new generation of cancer medications targeted to metabolic pathways. Without a scientifically sound neoplasm classification, biological measurements on individual tumor samples cannot be generalized to class-related tumors, and constitutive properties common to a class of tumors cannot be distinguished from uninformative data in complex and chaotic biological systems. This paper discusses the importance of biological classification and examines several different approaches to the specific problem of tumor classification.

## Background

Classifications provide a simplified view of a knowledge domain, with members of the domain grouped in a class hierarchy. Members of a class share one or more common features and inherit the class features of their ancestral classes [1,2]. Scientists use classifications to discover and test generalizable methods and properties that may apply to members of a class and their descendants. Much of the following discussion relating to the purpose and properties of classifications was inspired by Ernst Mayr, one of the leading evolutionary biologists of the past century [1]. Readers may have somewhat different views on the general subject of classification, but the views presented here are representative of a widely accepted approach developed by biologists.

The subject of tumor classification is made confusing by a variety of commonly held notions about the meaning and purposes of modern classifications [1]. Pathologists typically refer to anatomic tumor classifications when they are more accurately referring to lists of primary tumors that are known to occur at a particular location [3-7]. A list of tumors occurring at a body site is not a classification because it includes tumors that are biologically, clinically, and histologically unrelated. Although often referred to as World Health Organization (WHO) "classifications", the WHO accurately titles their organ-based lists of neoplasms as "Histologic Typings" for the different organs [8-12].

In the past decade, molecular biologists have tried to classify tumors based on grouping together tumor samples that share similar gene expression profiles [13-16]. The ability to separate tumors into groups is not equivalent to separating tumors into classes because the groups may represent expected variations of behavior within a single tumor population. For example, tumor samples of a particular type of tumor may contain groups that are separable based on proliferation rate, cell death rate, size, invasiveness, dominance of glycolytic enzyme pathways, etc. Variant groups within a population do not qualify as classes if it can be shown that the differences between the groups can be accounted for by transient differences in a tumor's biology. If a slow-growing tumor becomes a fast-growing tumor over time, or if a single tumor has foci of slow growth and fast growth, then the tumor cannot be classed by its rate of growth. A key principle in classification is that classes are intransitive (i.e. instances of a class never change their classs) [1]. Carcinomas never become lymphomas and lymphomas never become carcinomas. Grouping tumors by shared gene expression profiles may indicate that a certain tumor shares a similar profile with another tumor (for a chosen set of expressed genes), but it does not guarantee an intransitive classification of neoplasms.

**Figure 1:**
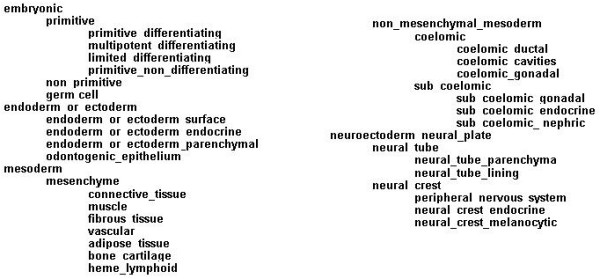
Developmental Lineage Classification Schema [2].

2004 marked the introduction of a classification of tumors based on developmental lineage [2] similar to that proposed by pathologists in the mid-20th century [17]. The rationale for the new classification is that tumor cells will tend to use metabolic pathways inherited from their ancestral cells within their developmental lineage [see: Figure 1]. As pathway-specific targets for cancer treatment become available for clinical trials, it may prove efficacious to test these agents on tumors that have a common lineage [18]. Also in 2004, a comprehensive taxonomy of neoplasms was created by expanding the NCI-Thesaurus [19-21]. The new taxonomy contains over 140,000 names of neoplasms and is included as a supplemental file with this manuscript [see Additional file 1]. This taxonomy was ported into the new classification of neoplasms to create "The developmental lineage classification and taxonomy of neoplasms," hereinafter called "The developmental classification"[19].

In addition to providing a useful nomenclature for neoplasms, the taxonomy reconciles differences between morphology-based classifications of neoplasms (favored by pathologists) and newly emerging classifications based on genomic characterizations of tumors [22-24]. Specific features of the developmental classification and taxonomy are [2]:

1. The classification is comprehensive (e.g. every tumor of man can be placed somewhere within the classification, which is the largest listing of neoplasm terms [19]. It is available at no cost in XML and flat-file formats. In either format, each term is annotated with its complete ancestral lineage. The most recent version of the nomenclature is made available at the Association for Pathology Informatics download page [25].

2. The classification is simple. One of the purposes of a classification is to drive down the complexity that exists when the domain taxonomy is large. The entire classification is described by under 40 classifiers.

3. The classification is based on biologic principle and is not determined by an artificial construct such as medical specialty (e.g. dermatologic neoplasms) or by anatomically vague regions (e.g. head and neck tumors) or by any single taxon (e.g. epithelial versus spindled morphology)

4. The classification is represented as an eXtensible Markup Language (XML) document that permits data integration between heterogeneous biomedical databases.

5. The classification does not invalidate existing diagnoses found in pathology reports. The medicolegal importance of this feature cannot be exaggerated. This relieves pathologists from reviewing all their prior cases and re-diagnosing them in conformance with a new classification.

The developmental classification is not based on morphologic or genomic properties of tumors. It is based on developmental lineage. However, the class instances (i.e., tumors) all have morphologic and genetic properties. Genetic changes that control particular pathways leading to a particular morphologic phenotype may have class specificity within the developmental classification [26]. Consequently, the developmental classification can be examined to determine how it deals with apparent discrepancies between the morphologic and molecular classifications of neoplasms.

In this study, six fundamental issues in tumor classification are described that exemplify the growing rift between morphologic and molecular approaches to tumor classification: 1) the separation between epithelial and non-epithelial tumors; 2) the grouping of tumors based on shared cellular functions; 3) the distinction between germ cell tumors and pluripotent tumors of non-germ cell origin; 4) the distinction between tumors that have lost their differentiation and tumors that arise from uncommitted stem cells; 5) the molecular properties shared by morphologically disparate tumors that have a common developmental lineage, and 6) the problem of re-classifying morphologically identical but clinically distinct subsets of tumors. The discussion of these issues in the context of describing different methods of tumor classification is intended to underscore the clinical value of a robust tumor classification.

The purpose of this manuscript is to show that grouping tumors according to their developmental lineage can reconcile certain fundamental discrepancies resulting from morphologic and molecular approaches to neoplasm classification.

## Discussion

### The separation between epithelial and non-epithelial tumors

The separation of tumors into two large groups, epithelial and non-epithelial, is a popular device [27-31]. It has certain practical advantages for clinicians. If a patient has a brain mass that is stereotactically biopsied, the pathologist can make a quick, intra-operative consultation based on a small sampling of the specimen, reporting that the tumor is epithelial or non-epithelial. If it's epithelial, the tumor is unlikely to be a primary brain tumor. It is much more likely to be a metastatic lesion from a primary carcinoma that arose somewhere else in the body. The treatment of a primary brain tumor is different from the treatment of a metastatic tumor to the brain. Hence, the intra-operative assessment of epithelial morphology can be very helpful to the surgeon.

Despite the clinical utility, the separation of tumors into epithelial and non-epithelial subtypes does not provide a consistent dichotomy for tumor classification. Experts in the field of classification consider it poor form to create a class based on criteria of exclusion. Classifications are intended to identify properties common to classes [1]. There is seldom much value in assigning class membership based on the absence of a property found in another class.

In the specific case of a morphologic separation of tumors into epithelial and non-epithelial classes, a problem arises when experts do not agree on the qualifications of epithelial tumors. Some might argue that epithelial tumors are tumors that arise from an epithelium (e.g., squamous cell carcinoma) or an epithelial glandular lining (e.g., adenocarcinoma). The qualifying features of an epithelium are controversial. Are mesothelial cells (which cover the serosal surfaces of the pleura and peritoneum) epithelial cells? Mesotheliomas arising from coelomic epithelium are classed as [non-epithelial] sarcomas. Are specialized mesothelial cells that cover the serosal surface of the ovaries epithelial? Ovarian tumors arising from specialized coelomic mesothelium, including papillary cystadenocarcinoma, are classed as epithelial tumors.

Some pathologists divide tumors into epithelial and non-epithelial classes based purely on morphology without regard to their tissues of origin. An epithelial cell is a round or polyhedral cell that adheres closely to other tumor cells with desmosomes (cell junctions). Pathologists subscribing to a purely morphologic separation of tumors face an assortment of problems when they try to maintain a dichotomous classification.

1. Some tumors are mixed epithelial and non-epithelial (mixed tumor of salivary gland, fibroadenoma of breast, synovial sarcoma, carcinosarcoma of uterus) [29,32].

2. Some tumors that are typically epithelial can have non-epithelial variants (e.g., sarcomatoid squamous cell carcinoma, spindle cell nevus, sarcomatoid melanoma).

3. Some tumors that are typically non-epithelial can have epithelial morphology (epithelioid leiomyoma) or morphologic features in common with epithelial tumors (clear cell sarcoma of soft parts) [33]

4. Biochemical markers intended to distinguish epithelial fron non-epithelial cells often co-express in epithelial and non-epithelial tumors [30].

Aside from the failure of the epithelial/non-epithelial dichotomy to provide a consistent basis for tumor classification, this approach to tumor classification creates discrepancies with molecular classifications of tumors. Genetic markers characteristic of tumors can occur in both epithelial and non-epithelial cancers. Furthermore, a single oncogene or molecular marker may characterize both epithelial and non-epithelial tumors [15,34,35]. This means that the epithelial/non-epithelial tumor dichotomy conflicts with a molecular classification of tumors.

The developmental classification assigns morphologically diverse tumors to a single class if they have the same developmental lineage. Morphologic attributes (such as epithelial or non-epithelial appearance) can be preserved as tumor annotations.

### Classifying tumors based on shared cellular functions

The endocrine tumors exemplify the confusion that occurs when tumors are classified by common function. Examples of endocrine organs include the thyroid gland and the adrenal gland. The U.S. National Cancer Institute lists 8 major endocrine glands and several other tissues that secrete physiologically active hormones [36].

Pituitary gland

Pineal gland

Thyroid gland

Parathyroid gland

Adrenal gland

Pancreatic islets of langerhans

Gonads (testes and ovaries)

Other endocrine glands – thymus, stomach, small intestines, heart, and placenta.

The problem with considering the endocrine tumors as a single class of tumors is that other than a common function (i.e. hormone secretion), tissues of the endocrine system my have nothing in common morphologically, genetically, or developmentally. Some of the endocrine glands have epithelial morphology (e.g. thyroid) others have spindle morphology (e.g. ovarian stroma). Any single endocrine gland is likely to contain several specialized cell types. The pituitary gland is divided into adenohypophysis (glandular part of the pituitary) and neurohypophysis (neural part of pituitary). The thyroid contains follicular cells and small nests of so-called C-cells that produce calcitonin. Thyroid follicular cells have an endodermal origin and thyroid C-cells have a neural crest origin. The adrenal gland has an epithelial cortex and a morphologically distinctive medullary portion. The adrenal cortex derives from mesoderm. The adrenal medulla consists of paraganglia cells derived from neural crest. As judged by embryologic origin and by morphology, the endocrine glands have no common [class] properties.

Within any endocrine organ are a variety of specialized cells that give rise to morphologically and clinically diverse neoplasms. The following list of tumors comprises a published "Classification of thyroid neoplasms" [37].

Follicular adenoma

Papillary adenoma

Atypical adenoma

Papillary carcinoma

Follicular carcinoma

Undifferentiated anaplastic carcinoma

Medullary carcinoma

Squamous cell carcinoma

Metastatic carcinoma

Fibrosarcoma

Angiosarcoma

Lymphoma

Teratoma

Other sarcomas

(oxyphil and clear cell variants listed as variants, not as members of the primary classification)

This classification of thyroid neoplasia includes tumors from several different lineages, including endodermal (e.g., follicular carcinomas of thyroid), neural crest (e.g. medullary carcinoma) mesenchymal (e.g., fibrosarcoma) and germ cell (e.g., teratoma). It seems unlikely that a single carcinogenic process would be responsible for the diverse tumors occurring in the thyroid or that a single metabolic pathway would provide a rational treatment target for all these tumors.

The advantages of classing endocrine neoplasms by lineage, rather than by function, become apparent when examining the co-occurrence of endocrine neoplasias in inherited tumor syndromes. Inherited tumor syndromes have germline aberrations that lead to multiple types of tumors occurring in affected individuals [38]. In many cases, the specific germline mutation or the involved metabolic pathway of an inherited syndrome has been characterized. Inherited disorders often involve tissues derived from a single developmental lineage (the "One disorder – One developmental lineage" rule). The following three generalizations demonstrate the concept:

1. Tumor syndromes that involve endocrine tumors and non-endocrine tumors recruit both endocrine tumors and non-endocrine tumors from a single developmental lineage

Example:

MEN2B – OMIM # 162300 (neural crest)

A single identical point mutation (exon 16) in the catalytic core of the tyrosine kinase domain of the ret gene has been found to be associated with both inherited and de novo MEN2B

Ganglioneuroma (ganglioneuromatosis);

Pheochromocytoma;

Calcitonin secreting medullary thyroid carcinoma;

*Parathyroid hyperplasia/adenoma (probably secondary to calcitonin secretion)

2. Tumor syndromes that exclusively involve endocrine tumors tend to recruit tumors from a single developmental lineage

Example:

MEN1 – OMIM # 131100 (endodermal origin)

The MEN1 gene contains 10 exons and encodes a ubiquitously expressed 2.8-kb transcript. The predicted 610-amino acid protein product is termed menin, a putative tumor suppressor protein inactivated by MEN1 mutations.

Pancreatic islet cell adenoma;

Parathyroid adenoma;

Pituitary adenoma;

Prolactinoma

Glucagonoma

Insulinoma

Vasointestinal peptide tumor

Gastrinoma

Carcinoid tumors

*Adrenocortical adenoma (secondary to Cushing syndrome [39])

3. Tumor syndromes associated with a mutation of a single metabolic pathway tend to recruit endocrine and non-endocrine tumors from a single developmental lineage.

Example:

Complex II mitochondrial pathway-associated tumors (neural crest [40])

Complex II, of which succinate dehydrogenase (EC 1.3.99.1) is a component, has 4 subunits:

The flavoprotein (SDHA; 600857),

No tumors, but is associated with Leigh syndrome of infantile subacute necrotizing encephalopathy [OMIM record 256000]

The iron sulfur protein (SDHB; 185470)

Carotid body tumors (cervical paraganglioma), and multiple extra-adrenal pheochromocytomas [41,42] [OMIM record 115310]

Pheochromocytoma [OMIM record 171300]

The 2 integral membrane proteins

SDHC (602413)

Familial nonchromaffin paragangliomas type 3 [OMIM recrod 605373][43]

SDHD (602690)

Paragangliomas, chemodectomas, carotid body tumors, glomus jugulare tumors [OMIM 168000]

Exceptions occur, and this is to be expected considering the multi-factor and multi-step nature of carcinogenesis. Tumors occurring in the von Hippel-Lindau syndrome (VHL) pose a seeming exception to the "One disorder – One lineage" rule. VHL is is a dominantly inherited familial cancer syndrome predisposing to a variety of malignant and benign neoplasms, most frequently retinal, cerebellar, and spinal hemangioblastoma, renal cell carcinoma, pheochromocytoma, and pancreatic endocrine tumors. It is caused by mutation in the VHL gene [OMIM record 608537]. VHL is characterized by tumors of the mesenchyme consisting of unusual angiomata, including angioma of retina and spinal cord, and hemangioblastoma of cerebellum. Also seen are pheochromocytomas (neural crest), renal cell carcinomas (mesoderm), and pancreatic islet cell tumors (endoderm). There are several possible explanations for this multilineage tumor syndrome. The mutation may involve a general cancer gene that triggers carcinogenic events independent of cell lineage. Alternately, VHL may be a complex disease that can involve multiple mutations targeting different developmental lineages. There is a tendency for specific combinations of tumors to cluster in different VHL families [44]. Both angiomatosis retinae and hemangioblastoma of the CNS occurred in most families, while renal cancer did not occur in VHL families with pheochromocytoma. This may mean that VHL is not a single mutational disorder. Perhaps the simplest hypothesis is that the VHL mutation targets [for carcinogenic transformation] a stem cell that precedes differentiation of the embryonic layers (endoderm, ectoderm, mesoderm and neuroectoderm). The tumors that occur in VHL families may therefore arise from any of the lineages that descend from the [hypothetical] VHL cancer stem cell.

A molecular classification of neoplasms tied to function (e.g., endocrine function) does not serve as a consistent class scaffold. Germline mutations that predispose to cancer do not select for target tissues that share a common function. Selection seems to favor tissues that share a common developmental lineage.

### The distinction between germ cell tumors and pluripotent tumors of non-germ cell origin

Nowhere is the dissonance between morphology, molecular biology and developmental biology more striking than in the realm of the germ cell tumors. The germ cell tumors include tumors of diverse morphology, including seminomas (male), dysgerminomas (female), teratomas, embryonal carcinomas, endodermal sinus tumors, and some (but not all!) cases of choriocarcinoma [45]. Germ cell tumors also have diverse molecular markers with cytogenetic changes correllating with age of patient rather than type of germ cell tumor. Pediatric germ cell tumors show imbalances in of chromosome 1 and loss of 6q, while adult germ cell tumors often have an isochromosome 12p or amplification of 12p [45]. Germ cell tumors arise in almost any part of the body (particularly midline locations) and any age (with a pediatric dominance). It was difficult to imagine a way of placing all these tumors into a single class of neoplasms until Teilum suggested that all these tumors had the same cell of origin, the primordial germ cell [46]. The biologic mechanism by which one cellular progenitor can give rise to these seemingly unrelated tumors is still unfolding as a fascinating saga of developmental biology [45].

Germ cells normally follow a narrow developmental path. Male germ cells give rise to spermatocytes. Female germ cells give rise to oocytes. Under normal circumstances, germ cells are not pluripotent and are not related to embryonic stem cells. The only tumors arising directly from neoplastic germ cells are pure seminomas and dysgerminomas (the female equivalent of seminomas). At an early embryonic stage, primordial germ cells undergo a complete erasure of epigenetic programming, a phenomenon that uniquely characterizes these cells. This phenomenon precedes the transformation of primordial germ cells into embryonic stem cells in culture, is the putative mechanism that operates in animals to provide neoplastic precursors of totipotent tumors, and provides a biological rationale for separating germ cell tumors from other embryonic tumors [47-49]. It permits us to think of germ cells and their normal descendants (ova and sperm) as tumors occupying their own sub-class. We can think of the totipotent and embryonic tumors as tumors of embryonic stem cells (not germ cells). These embryonic stem cells may have derived from the phase of germ cell development when epigenetic programming is erased [48,49], but the resulting cells can be separately classified based on their biology and their shared epigenetic properties characteristic of a totipotent phenotype. By separating tumors of embryonic cells from tumors derived from germ cells, certain incongruities are avoided. In the developmental classification, a gestational choriocarcinoma (arising from cytotrophoblasts and syncytiotrophoblasts in the placenta and having no derivation from germ cells) can now be classed separately from seminomas. Furthermore, new drugs that target the germ cell tumors may find that differences in the genomic state of germ cell tumors and embryonic tumors (i.e., methylation patterns) may uncover vulnerabilities that provide new treatment options. Finally, a developmental classification that distinguishes germ cell tumors and embryonic tumors reconciles the fundamental differences between the morphologic and cytogenetic incongruities among these unique tumors.

### The distinction between tumors that have lost their differentiation and tumors that arise from embryonic stem cells

Tumors that have grown into large tumor masses that deeply invade surrounding tissues and metastasize to many distant organs tend to have different morphology than tumors that are small and pre-invasive. Tumors in so-called late lesions tend to acquire morphologic features that are not found in non-neoplastic cells. The nucleus is markedly different from the normal nucleus with angulations of the nuclear membrane, splotchy chromaticity within the nucleus, and marked variation in nuclear size and shape from one tumor cell to the next. Cellularity is often high (i.e. more cells in a microscopic field compared with normal tissues). Mitotic figures, which may be abundant in a late tumor, tend to have abnormal mitotic spindles. Cytogenetics performed on late tumors often show aneuploidy with multiple, complex karyotypic abnormalities. In general, late lesions of widely different tumor types often resemble one another more than they may resemble the early cancer from which they arose. Pathologists often refer to tumors that have lost differentiated morphologic features [found in early lesions] as undifferentiated or poorly differentiated malignancies, implying that the tumor cells have lost properties characteristic of differentiated cell lineages.

A number of tumors, particularly in childhood, consist of cells that have no apparent developmental cell lineage. The small round cell tumors of childhood are prototypical examples. These tumor cells tend to have a uniform size within the tumor. They tend to be characterized by very specific balanced translocations leading to the creation of specific fusion genes [50]. These tumors are sometimes referred to as undifferentiated or primitive neoplasms.

The pathologist's concept of an "undifferentiated" tumor has been applied to such biologically diverse tumors as Ewing's tumor and late stage colon carcinomas. This exemplifies the limitations of grouping tumors using a morphologic feature (such as loss of differentiation) that does not distinquish pathogenetic mechanisms. By classifying tumors by their developmental lineage, useful morphologic features (including extent of differentation) can be applied without obscuring the class features that define the different tumor types.

### The relationship among morphologically disparate tumors that share a developmental lineage

Epithelial tumors of the kidney and of the uterus have biologic features that separate them from epithelial tumors in other organs. Furthermore, many of the properties of epithelial tumors arising from kidney or from uterus are specifically associated with mesenchymal tumors.

The gene expression profiles of renal epithelial tumors are much more closely related to the gene expression profiles of sarcomas than of epithelial tumors derived from other organs [15]. Several epithelial tumor variants are characterized by specific tranlocations resulting in fusion genes [34,35,51]. Fusion genes are much more characteristic of sarcomas than epithelial tumors [24]. Mesoblastic nephroma, shares an identical fusion marker (TV6- NTRK3) with congenital fibrosarcoma [52]. Several variants of renal epithelial tumors have the same gene fusion marker as alveolar soft part sarcoma [34,35]. A diverse array of epithelial, stromal and mixed epithelial-stromal tumors are known to arise from kidney parenchyma [53]. How is it possible for an epithelial organ to give rise to epithelial and stromal tumors with epithelial tumors characterized by sarcoma molecular markers?

The kidney is an epithelial organ that has mesodermal lineage. No cells in the kidney arise from endoderm or ectoderm, the major embryonic lineages that give rise to most epithelial tumors. In a sense, the kidney is a stromal organ that masquerades as an epithelial organ. The only way to reconcile the discrepancy between morphologically epithelial renal tumors and their sarcomatous molecular features is to recognize that renal tumors can be classed according to their mesodermal lineage [2].

A similar story holds for uterine tumors. The uterus gives rise to adenocarcinomas, sarcomas, and variously named mixed tumors including carcinosarcomas, adenosarcomas, and mixed mullerian tumors with heterologous components. The histogenesis of mixed epithelial and stromal tumors of the uterus has always presented a special intellectual challenge. Both epithelial and non-epithelial tumors of the uterus seem to have an association with tamoxifen therapy [54], suggesting the possibility that a single stem cell targeted by tamoxifen can give rise epithelial and stromal tumors of the uterus.

In most other organs, epithelial cells do not share their genesis with mesenchymal cells. But the uterus, like the kidney, is derived entirely from mesoderm. The uterus is formed from a duct that forms within the mesoderm (the paramesonephric duct). This duct gives rise to the endometrial epithelium as well as the underlying stroma. Consequently, tumors of endometrial and stromal cells share the same lineage in the developmental classification (sub-coelomic ductal). Like the kidney, this classification ignores morphologic differences (epithelial versus mesenchymal) and creates a grouping in concordance with the observed mixed epithelial/stromal manifestations of some uterine tumors. This is another example wherein the developmental classification accommodates morphologic class ambiguity.

### The problem of determining when morphologically identical but clinically distinct subsets of tumors constitute new slots in the classification

Whenever new subpopulations of tumors can be delineated, the question of classification arises. If a report shows that a fraction of a certain type of tumor has a special morphologic feature, does the morphologic variant qualify as a new subclass of the tumor? If a tumor can be divided into two distinct clusters based on gene expression profiles, is each cluster a new subclass of the tumor? If patients with a certain tumor can be divided into good prognosis and bad prognosis groups, can the tumor be subclassified based on clinical prognosis? The problem with classifying based on morphologic, molecular or clinical features is that any population can be expected to contain members with features that vary from the population norm. There is a difference between segregating population variants (e.g. fast runners and slow runners, chocolate lovers and chocolate haters) and creating a self-consistent classification. A classification has certain properties that distinguish it from other ways of organizing data. The general rules for classifications have been summarized [1,2]:

1. A classification is a hierarchical grouping, with each group defined by the greatest number of taxa (informative features) that can apply to every instance of the class.

2. Subclasses inherit the properties (shared taxons) of their ancestor classes.

3. Every instance of the knowledge domain must fit into the classification, and every instance and class must have exactly one slot in the classification.

4. Instances of one class are intransitive (e.g. an instance in one class cannot migrate to a different class, but must remain in the same class or a subclass of the same class).

5. A classification is a hypothesis about the fundamental properties of a knowledge domain. The hypotheses must be tested and re-tested and changed when the facts do not fit the model.

If a tumor lacks a morphologic or molecular feature at one point in its development and gains the feature at a later point, the feature cannot determine a new class of tumor. If a tumor has a good prognosis at one point (e.g. before it has metastasized) and a bad prognosis later (e.g. after it has metastasized), then prognostic features associated with metastasis cannot be used to determine a new class of tumor. In general, all new findings about subpopulations of tumors can be considered candidate taxa (i.e., features that characterize a class and distinguish the class from other classes). The full list of class features (items 1–5 above) must be satisfied before a candidate taxon can be used to define a new tumor class.

### The future role of morphology and molecular analysis in tumor classification

Morphologic pathology has dominated tumor diagnosis for over 150 years [55]. The demise of morphologic pathology is a long-anticipated event that may never occur. In fact, the morphologic pathologist is a key developer of emerging technologies that promise the end of our dependence on histologic evaluation of lesions. It is the pathologist who prepares, describes and diagnoses the tissue samples used by the molecular biologist. It is the pathologist who collects, organizes and integrates the information in the patient's surgical pathology report with demographic information (age, gender), medical history, tumor staging, and ancillary hospital tests (radiology reports, hematology reports, and past/future tissue reports). In most cases, researchers would have no tumor samples for gene expression profiling if the pathologists had not been able to distinguish the tumor samples by careful morphologic evaluation. In all cases, the clinico-pathologic annotations used by the molecular biologist are generated in whole or in part by surgical pathologists. It has been noted that, "the pathologist's understanding of anatomic, physiologic, biochemical, immune, and other underlying factors that drive mechanisms of tissue responses to noxious agents turns a bewildering array of gene expression data into focused research programs"[56].

If molecular classification is to replace the morphologic classification of tumors, several seemingly intractable problems must be solved. Cytogenetic abnormalities and gene alterations in tumors co-occur with other abnormalities, and the complex state of molecular abnormalities in tumors makes it very difficult to settle on a set of alterations characteristic of classes of tumors. As an example, balanced translocations play biologic roles in several dozen tumors [24]. Although certain translocations are characteristic of individual tumors, it has proven difficult to generalize that translocations occur in any particular class of tumors. Certainly, characteristic tumor translocations occur more commonly in mesenchymal tumors [24], but such translocations have also been observed in secretory carcinoma of breast [57] and in midline [lung] carcinoma of children and young adults [58]. The notable exception wherein a class of tumors is characterized by a set of translocations is the Ewing's tumor family of tumors [50].

Mitelman has argued that translocations are tissue non-specific, occurring at a frequency related to the overall number of cytogenetic abnormalities found in tumors [59]. If it is difficult to assign classes of tumor to a single type of cytogenetic abnormality, it may be impossible to reach scientific consensus on complex sets of molecular signatures that define groups of tumors. It can be noted that despite numerous projects aimed at classifying tumors with gene expression profiles, no comprehensive classification based on this technology has emerged.

Much of what passes for neoplasm "classification" in the bioinformatics literature is actually the algorithmic ranking of expressed genes that can discriminate one tumor variant from another [13,14,60]. Once candidate molecules (i.e., genes, proteins, and other macromolecules or patterns of these molecules) are found to associate with a particular tumor variant, the pathologist gets a second chance to determine if a morphologic pattern correlates with the molecular property. An example comes from the study of gastrointestinal stromal tumors (GIST). Most GIST tumors have a c-Kit mutation that results in c-Kit protein overexpression [61]. Some GIST tumors lacking c-Kit mutations have a mutation in the platelet-derived growth factor receptor alpha gene [62]. Sakurai and coworkers have examined GIST tumors that stain negatively for CD-117, a marker for c-Kit protein overexpression. Many of these tumors have mutations in platelet-derived growth factor receptor alpha gene and a distinctive histomorphology characterized by myxoid epithelioid tumor cells and tumor infiltration by mast cells [63]. This newly recognized subtype of GIST involved the morphologic re-examination of the tumors following a molecular discovery.

Other examples abound. Secretory carcinoma of breast is an uncommon variant of breast cancer that occurs most frequently in young women. It is characterized by the ETV6-NTRK3 fusion gene [57]. The search and discovery of this molecular marker was accomplished through asynchronous contributions from three biomedical realms: 1) pathologists, who found defined the morphologic subset of breast carcinoma known as secretory carcinoma of breast; 2) oncologists who validated the clinically distinct features of the tumor, and 3) molecular biologists who discovered the translocation that characterized the tumor.

It is a basic assumption of the developmental classification that morphologic and molecular features of tumors will both fall sensibly into classes determined by tumor cell lineage. It is further assumed that pathways with molecular alterations producing a tumor phenotype will tend to operate in all tumors of a developmental class. Finally, it is hoped that morphologic properties associated with the altered pathway will be visible in all class members. Because classifications are hypotheses about the fundamental nature of a knowledge domain, the foundational assumptions of any classification must be continually evaluated and challenged.

Some classifications can be challenged more easily than others. A classification built on a set of continually changing parameters is constantly changing and difficult to evaluate. This is certainly true of a molecular classification, because our knowledge of the field changes almost daily. A few years ago, it was safe to say that all recurrent balanced translocations were a phenomenon of mesenchymal tumors. New findings of recurrent balanced translocations in non-mesenchymal tumors have nullified this class assertion [59]. Morphologists once classified clear cell sarcoma as a type of malignant melanoma, based on finding melanosomes within tumor cells. Recent molecular classification of these tumors clearly distinguish them from cutaneous melanoma. Clear cell sarcomas have characteristic EWS-AFT1 fusion transcript not found in cutaneous melanomas [64]. In addition, BRAF mutations, commonly found in cutaneous melanomas, are absent from clear cell sarcomas [65]. The rapid accumulation of new knowledge about the molecular characteristics of tumors can quickly change classifications built on morphology or molecular biology. Pathologists seem to be putting this tumor back into the mesenchymal class of neoplasms [66].

The developmental classification is built on a foundation of developmental biology that was improved over many decades by thousands of scientists. Our understanding of embryologic lineage has changed very little over the past half century, and a classification based on developmental biology permits tumors to be assigned to well-defined classes. Recent advances in embryology have shown that somatic DNA has lineage-specific epigentic modifications that occur throughout development [47,67]. This means that the developmental lineage of tumors may be measurable and refinable with new techniques that correlate patterns of epigenetic modifications (e.g. methylation) with lineage. In a recent paper by Kho and coworkers [68], the authors developed a method that projects gene expression profiles of tumors onto a mouse developmental sequence. Human medulloblastoma most closely matched the gene expression profile of postnatal day 5 mouse cerebellum. Although this study examined only a few tumors, it described a method that allows any human tumor to be matched against a library of gene expression profiles collected from normal tissues at different stages of development.

## Summary

A scientifically sound classification of neoplasms will serve as a guide to selecting a new generation of cancer medications targeted to metabolic pathways specific for particular classes of tumors. Without a classification of tumors, biological measurements on individual tumor samples cannot be generalized to other tumors, and constitutive properties common to a class of tumors cannot be distinguished from uninformative data collected from a complex and chaotic biological system. Morphology, even in the post-genomic era, has enormous value in the realm of taxon discovery. Using morphologic examination, pathologists have discovered previously unrecognized morphologic features that are diagnostic for new tumors or new clinical variants of known tumors that have characteristic molecular profiles. By classifying tumors by lineage, problems arising from molecular and morphologic tumor classifications can be resolved or posed as testable hypotheses.

## Competing interests

The author(s) declare that they have no competing interests.

## Pre-publication history

The pre-publication history for this paper can be accessed here:



## Supplementary Material

Additional File 1 **Neoplasia classification structure (XML version) **Neoclxml.gz is a compressed (gzipped) XML file. The downloaded file should be renamed neoclxml.gz so that the .gz suffix can be recognized by unzip utilities. Unzip the file (using a free, open source utility such as gunzip.exe [69], or a proprietary utility such as Winzip). Once unzipped, the file should be renamed neocl.xml, so that it will have an .xml suffix. If the file is too large for viewing on your web browser, it can be viewed on plain-text word processors. Click here for file
